# Examination of particulate matter concentrations of the outdoor air of Miercurea Ciuc, Romania

**DOI:** 10.1038/s41598-025-04528-w

**Published:** 2025-07-02

**Authors:** Katalin Bodor, Boglárka Geréd, Levente Szép, Levente Simó, Mátyás Gál, Zsolt Bodor

**Affiliations:** 1 Department of Food Engineering, Faculty of Economics, Socio-Human Sciences and Engineering, Sapientia Hungarian University of Transylvania, Libertății Sq. 1, 530104 Miercurea Ciuc, Romania; 2 Technology School “Venczel József”, st. Toplita 20, 530241 Miercurea Ciuc, Romania; 3 Department of Bioengineering, Faculty of Economics, Socio-Human Sciences and Engineering, Sapientia Hungarian University of Transylvania, Libertății Sq. 1, 530104 Miercurea Ciuc, Romania; 4 Research and Development Institute for Wildlife and Mountain Resources, st. Progresului 35B, 530240 Miercurea Ciuc, Romania

**Keywords:** Urban air quality, Road traffic emissions, Diurnal variation, Meteorological influence, Rainfall impact, Environmental monitoring, Environmental sciences, Risk factors

## Abstract

This research aimed to measure particulate matter concentrations (PM_1_, PM_2.5_, PM_4_, PM_10_, and total suspended particles) at five locations in Miercurea Ciuc, while also monitoring road traffic intensity and meteorological parameters. Significant differences in particulate matter concentrations were observed across various points in the city. On weekdays, morning aerosol levels were notably higher than those in the afternoon, whereas weekend concentrations were generally lower and more stable throughout the day. Early morning measurements showed a strong correlation with peak traffic periods. During rainy conditions, PM_10_ levels returned to baseline values. In the city center, PM_10_ concentrations were typically higher (93.16% of the time) than those recorded by the Environmental Protection Agency, averaging 3.27 times greater than at the HR_02_ station.

**Supplementary Information:**

The online version contains supplementary material available at 10.1038/s41598-025-04528-w.

## Introduction

Particulate matter (PM) concentration is one of the most critical factors in calculating air quality indices. PM is a complex mixture of organic and inorganic pollutant species that originate from both natural and anthropogenic sources. The emission source and health impact of PM are closely related to particle size, which is classified based on aerodynamic diameter. PM_10_ and PM_2.5_ represent the mass concentrations of particles with diameters of 10 µm and < 2.5 µm, respectively. The 2.5–10 µm particles are classified as coarse particulates (PM_10–2.5_), while those smaller than 2.5 µm are referred to as fine particulates due to their ability to accumulate in human organs; therefore, they have a significantly higher negative effect on human health^1^. Particulate matter smaller than 2.5 µm has its ability to bypass the natural defense mechanisms of the respiratory system. Unlike larger particles, which are often trapped in the nasal passages or upper airways, PM_2.5_ can penetrate deep into the alveoli of the lungs, where gas exchange occurs and subsequently enter the bloodstream. From there, these particles can reach critical organs such as the heart, liver, and even the brain. Once in the bloodstream, PM_2.5_ can trigger systemic inflammation and oxidative stress, which are linked to a range of chronic and acute health conditions. For example, exposure to fine particulate matter has been strongly associated with an increased risk of respiratory diseases such as asthma, bronchitis, and chronic obstructive pulmonary disease (COPD). Cardiovascular diseases, including ischemic heart disease, arrhythmias, and strokes, are also common outcomes of long-term PM_2.5_ exposure^2,3^.

The negative implications of PM pollution extend far beyond public health. It contributes to climate change by affecting the Earth’s radiation balance through light absorption and scattering, hence influencing weather patterns and global temperatures. Moreover, PM pollution can degrade ecosystems, reduce biodiversity and contaminate soil and water resources ^4^.

Urban air quality is affected by increasing traffic density, making air quality indices in city centers typically worse than those in suburban or rural areas. As a result, people living in cities receive a much higher dose of air pollutants, leading to more significant health risks^5^. Traffic restrictions and management measures are necessary to reduce the risk^6^. Ingestion, inhalation, and dermal absorption of particulate matter and its different fractions can cause several diseases, including cancer, respiratory disease, and cardiovascular disease^7,8^. According to the WHO Air Quality Guidelines, recommended PM_10_ concentration limits are as follows: annual mean ≤ 15 μg m^–3^ and daily mean ≤ 45 μg m^–3^ , and those for PM_2.5_ annual mean ≤ 10 μg m^–3^ and daily mean ≤ 25 μg m^–3^ ^9^.

In this study, we investigated the spatial and temporal evolution of aerosol concentrations in the outdoor air of Miercurea Ciuc.

The objectives of the multi-stage study were as follows: (1) To measure aerosol concentrations at five different locations in the city during weekdays and weekends, in both morning and afternoon hours. (2) To measure early morning particulate matter concentrations and simultaneous traffic intensities at different sampling points. (3) To conduct two weeks of continuous measurements in the city center. (4) To determine the washout effect of precipitations by analyzing meteorological parameters. (5) To compare the measured particulate matter concentrations with airborne dust levels reported by the National Environmental Protection Agency. (6) To calculate relative health risks and generate a summary relative risk table based on potential annual exposure concentrations.

## Materials and methods

### Study area

**Fig. 1:**
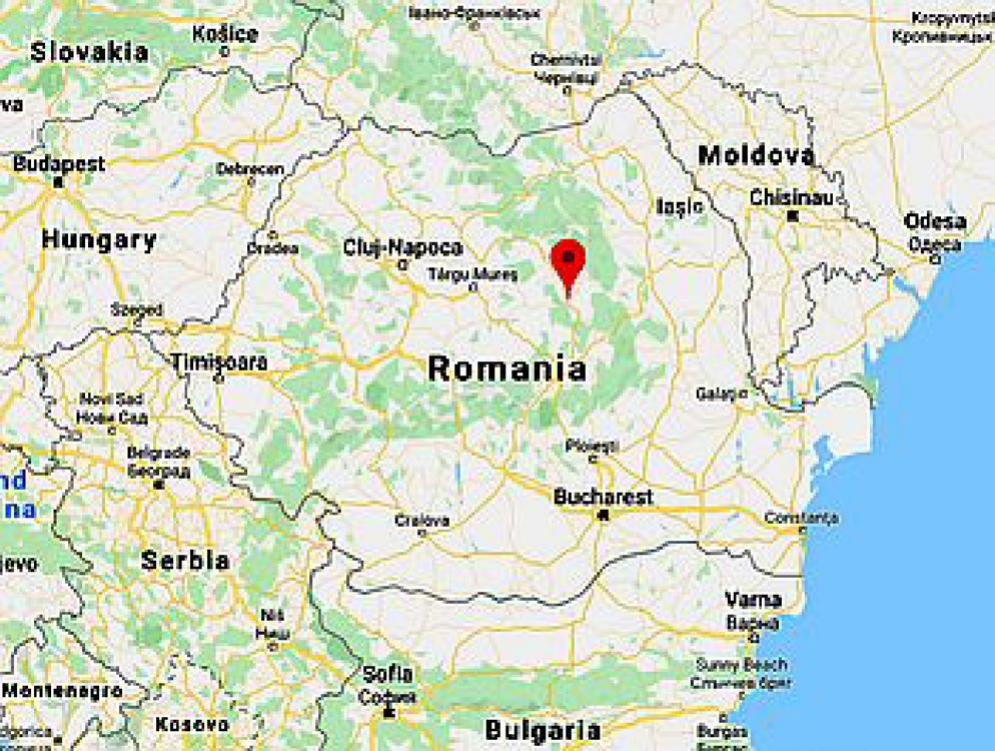
The sampling sites (Ciuc Basin)^10^.

Miercurea Ciuc is a Romanian city located in the Ciuc Basin in the eastern Carpathians. The Ciuc Basin is surrounded by mountains where thermal inversion and dense fog formation are common, especially during the cold period (December–March). Five different locations were selected as sampling points: 1. Libertății Sq, 2. Kaufland supermarket, 3. Nest Shopping Center, 4. Palace of Justice, 5. Railway station (Fig. 1).

The geographical parameters are listed in Table 1. The altitudes of the sampling points ranged from 659 to 697 m. For the comparative analysis, two monitoring stations (HR_01_ and HR_02_) were used for daily meteorological data and particulate matter concentrations.

**Table 1:** Geographical characteristics of the measuring points.

Sampling locations	Altitude, m	Latitude	Longitude
1. Freedom Square,	664	46.361	25.800
2. Kaufland supermarket,	659	46.366	25.797
3. Nest shopping center,	699	46.362	25.811
4. Palace of Justice,	681	46.355	25.803
5 Railway station	665	46.359	25.793
HR_01_ Monit. station	697	46.33	25.81
HR_02_ Monit. station	687	46.35	25.81

### Particulate matter measurements

**Fig. 2:**
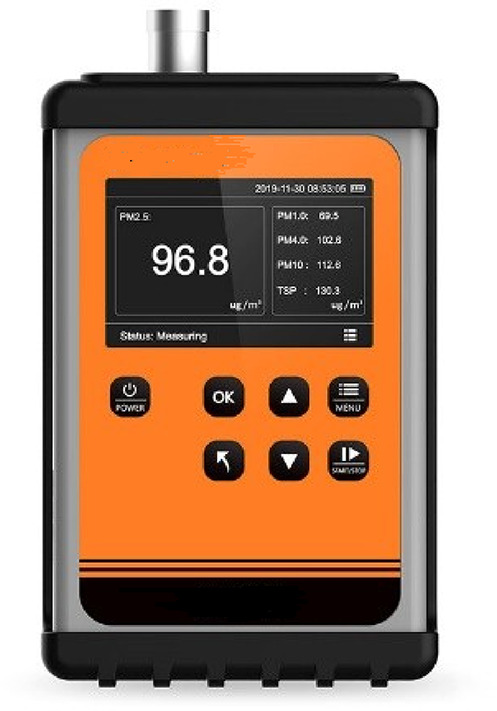
The Temtop PMD 351 air quality monitor used for PM measurements.

Particulate matter concentrations in Miercurea Ciuc (Romania) were monitored from October to November 2023. The measurements were carried out using a Temtop PMD 351 instrument, which recorded concentrations of PM_1.0_, PM_2.5_, PM_4.0_, PM_10_ and TSP (total suspended particles) fractions. The measurement range of the instrument was 0–1000 μgm^–3^ (Fig. 2).

 Accurate readings can be guaranteed within a temperature range of 0 °C and 50 °C. Additional details are available in Appendix Table S1. Measurements were taken at a distance of 4–6 meters from the roadway, with the instrument positioned 1.5 meters above ground level to simulate human exposure as accurately as possible—particularly the inhalation and ingestion levels of pedestrians. For comparative purposes, air pollutant concentrations and meteorological data were obtained from the HR_01_ and HR_02_ monitoring stations, operated by the National Environmental Protection Agency ^11^.

Particulate matter concentrations were evaluated through multiple steps:

1. Point measurements were conducted at five different locations on both weekdays and weekends, during morning and afternoon hours.
2. Continuous measurements were taken at various locations between 6:30 and 9 a.m. to examine the correlation between particulate matter concentrations and morning vehicle traffic.
3. In the city center, continuous measurements were performed from 7:00 a.m. to 7:00 p.m. over a two-week period.

### Particulate matter and climatological parameters

Among the meteorological parameters, hourly air temperature, precipitation, relative humidity, and the lifting condensation level (LCL) were monitored. During the study period (October–November 2023), hourly climatological data from the HR_01_ and HR_02_ monitoring stations were used to determine the LCL.

The lifting condensation level was estimated using air temperature and relative humidity, based on Eq. 1 and Eq. 2.

1 $$LCL = {z^0} + 125*\left( {T - {T_d}} \right)$$

2 $${T_d} = T - \left( {100 - RH} \right)/5$$

where: *LCL*—is the lifting condensation level (m), *T*—is the air temperature (°C), *RH*—is the relative humidity (%), *z*^*0*^—is the altitude (2 m), and *T*_*d*_—is the dew point temperature (°C) ^12^.

### Estimation of the precipitation washout effect on particulate matter

To assess the atmospheric cleansing effect of precipitation, daily particulate matter concentrations (PM_1_, PM_2.5_, PM_4_, PM_10_, and PTS) were compared between rainy and non-rainy periods. To evaluate wet deposition, three rainfall intensity categories were defined: I. low: 0.2–0.4 mm h^-1^, II. medium: 0.4–3.9 mm h^-1^, III. heavy rains: > 3.9 mm h^-1^).

### Study of the health-damaging effect of particulate matter

#### Health risk assessment methodology for short-term exposure to PM_10_

To assess the health risk associated with short-term exposure to PM_10_, the relative risk (RR) of all-cause mortality was calculated using Eq. 3, based on the methodology proposed by Ostro^13^. The calculation was applied when the average annual concentration of PM_10_ exceeded the background level (defined as 10 gm^–3^). The coefficient of the applied risk function was 0.008 (95% CI: 0.0006–0.0010).

3 $$RR\, = \,exp\left[ {\beta \left( {X--{X_0}} \right)} \right]$$

where: *X*_*0*_—is the background PM_10_ concentration (10 g m^–3^), *X*—is the annual average PM_10_ concentration (g m^–3^), and *β*—is the risk function coefficient.

#### Health risk assessment methodology for short-term exposure to PM_2.5_

Equation 4 was used to calculate the relative risk (*RR*) attributable to fine particulate matter (PM_2.5_). Risk-function coefficient (*β*) for specific health outcomes are available in the literature. In this study, we estimated the *RR* of mortality from lung cancer and cardiopulmunary diseases among residents aged > 30 years, using the coefficients reported by Ostro^13^.

4 $$RR \, = \, {\left[ {\left( {X \, + \, 1} \right)/\left( {{X_0} + \, 1} \right)} \right]^\beta }$$

where: *X*—is the annual average concentration of PM_2.5_ (µg m^–3^), *X*_*0*_—is the background concentration of PM_2.5_ (3 µg m^–3^), and *β*—is the coefficient of the risk function.

The *β* coefficient for cardiovascular disease was 0.15515 (95% CI 0.0562–0.2541), while the *β* coefficient for lung cancer was 0.23218 (95% CI 0.08563–0.37873).

To assess the distribution of the data, normality was evaluated using histograms. Spearman correlation coefficients were calculated between hourly PM_10_ concentrations and selected climatological parameters, including precipitation, air pressure, solar radiation, relative humidity, air temperature, and lifting condensation level (LCL). A total of 336 h of data were analyzed from the HR_02_ monitoring station, while 161 h were used from the city center location.

## Results and discussion

### Distribution of particulate matter

**Fig. 3:**
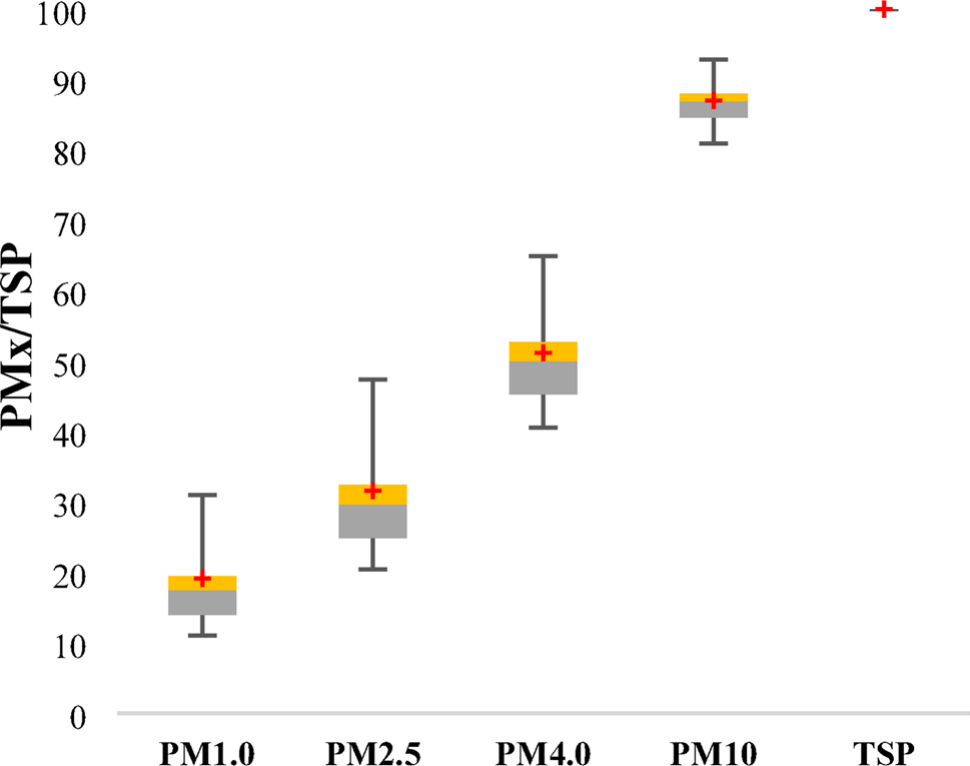
Particulate matter distribution compared to the TSP concentration.

Analysis of particulate matter distribution in the city center revealed that, on average, 87% of total suspended particles (TSP) consisted of aerosols with a diameter of 10 μm or less (PM_10_). The remaining 13% was composed of coarse particles larger than 10 μm Within this fraction, fine particulate matter (PM_2.5_) accounted for 31.6% of all TSPs (Fig. 3.).

### Particulate matter spatial distribution at five different points in the Miercurea Ciuc city

**Fig. 4:**
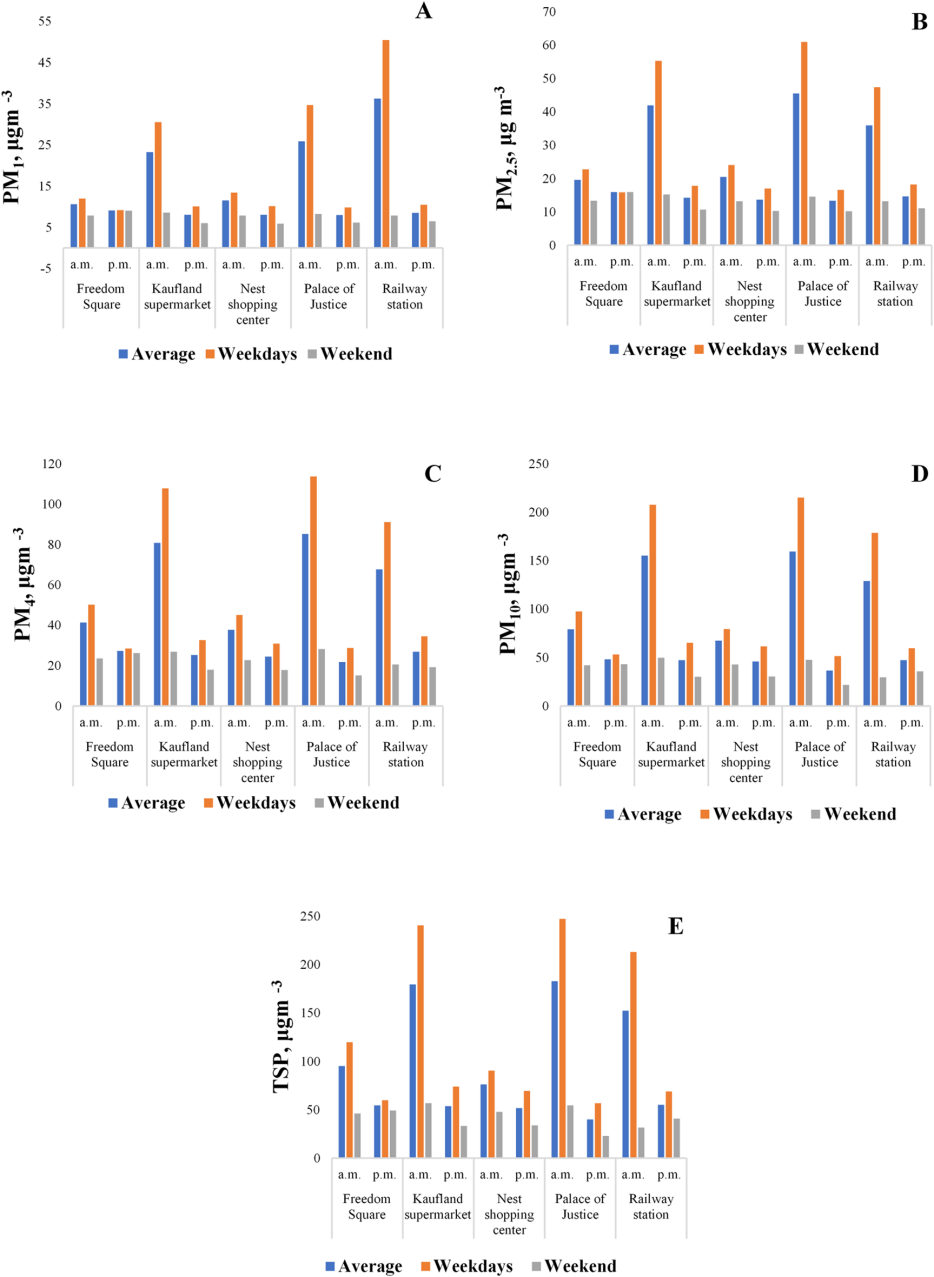
Spatial distribution of particulate matter (A-PM_1_, B-PM_2.5_, C-PM_4_, D-PM_10_, E-TSP.

Figure 4. summarizes the differences in particulate matter concentrations between the five sampling locations, comparing morning vs. afternoon and weekday vs. weekend values.

Based on the total suspended particulate (TSP) concentrations, the highest levels were detected at the Palace of Justice (1247.15 μg m^–3^) and Kaufland supermarket at (240.15 μg m^–3^) during weekday mornings. These were followed by the Railway station, with a concentration of 212.90 μg m^–3^. The maximum TSP concentrations at Freedom Square and the Nest shopping center were 119.75 μg m^–3^ and 90.40 μg m^–3^, respectively. The average TSP concentrations at the measurement sites on weekday mornings followed this decreasing order:

Palace of Justice 182.88 μg m^–3^ > Kaufland supermarket 179.31 μg m^–3^ > Railway station 152.45 μg m^–3^ > Freedom Square 95.16 μg m^–3^ > Nest shopping center 53.6 μg m^–3^.

At all five locations, the concentrations of each measured particulate matter fraction were consistently higher in the morning than in the afternoon. Weekday variability was most pronounced at Kaufland supermarket, the Palace of Justice, and the Railway station. For PM_1_, the morning concentration near Kaufland was 3.02 times higher than in the afternoon, while at the Palace of Justice, the difference was 3.53 times. The largest variation was observed at the Railway station, where the morning concentration was 4.8 times higher than the afternoon value. On weekends, morning and afternoon concentrations were closer, with less pronounced differences across all locations. For all five investigated particulate matter fractions (PM_1_, PM_2.5_, and PM_4_, PM_10_, TSP), similar and nearly identical distribution patterns were observed (Fig. 4).

**Fig. 5:**
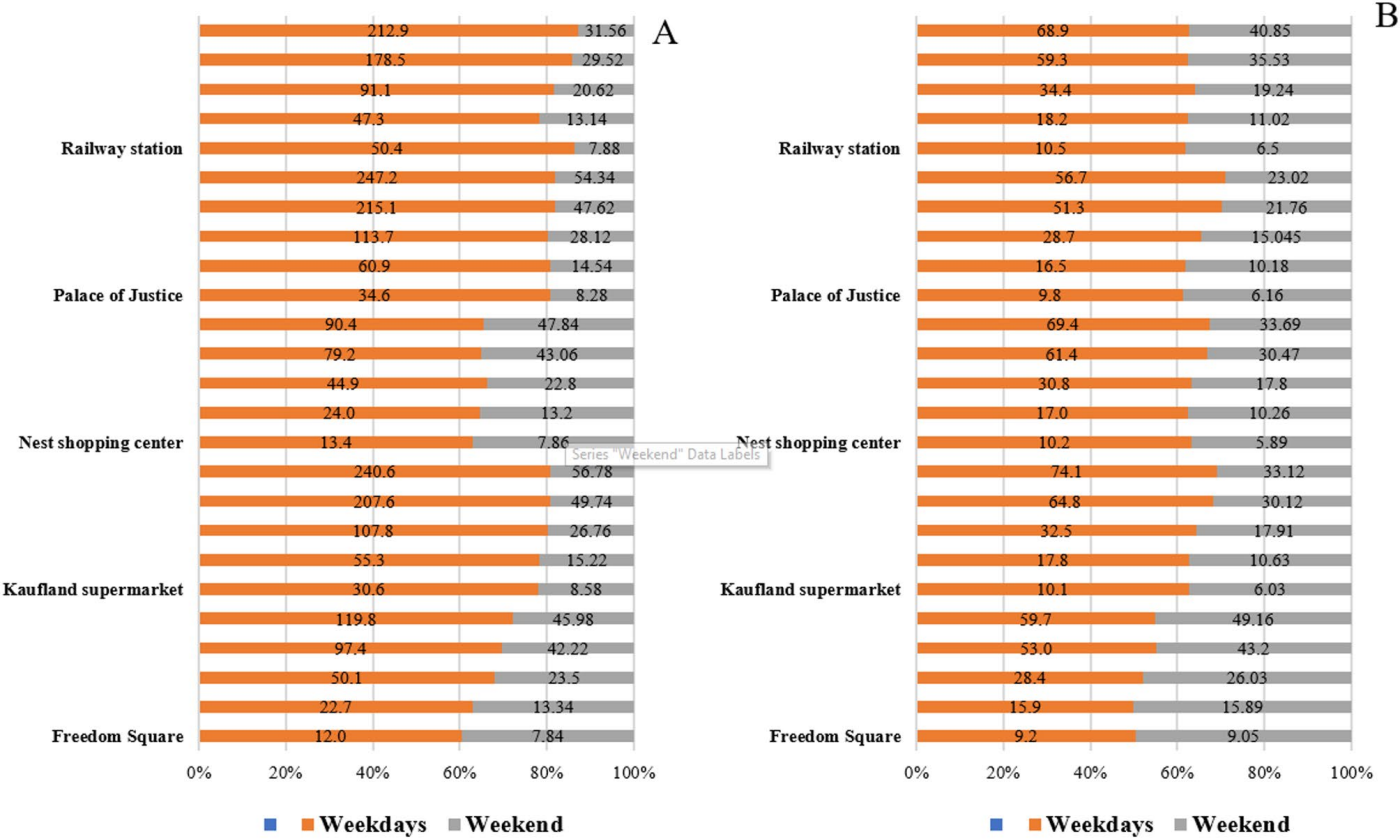
Evolution of the morning (**A**) and afternoon (**B**) particulate matter concentrations.

A significant difference was found between weekday and weekend particulate matter concentrations. At the Railway station, Kaufland, and the Palace of Justice, the weekday/weekend distribution was approximatively 80%/20%, indicating that weekday concentrations were four times higher than weekend levels. At the Nest shopping center, the weekday/weekend distribution was 66%/34%, reflecting a twofold difference. For Kaufland, the ratio was 77%/23%, representing a threefold decrease in particulate matter concentrations on weekends (Fig. 5.).

### Evaluation of climatological parameters

**Fig. 6:**
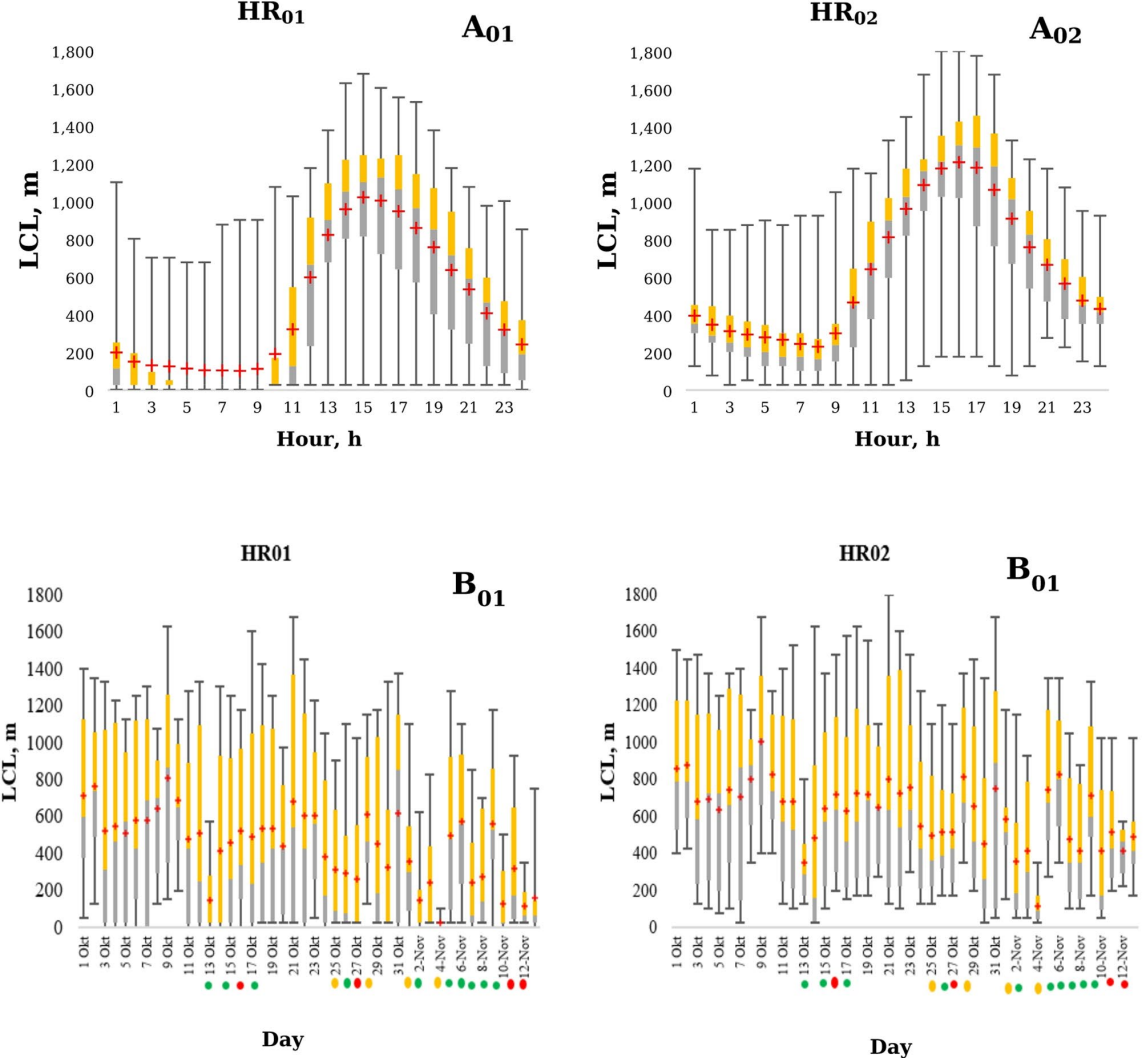
Evolution of the hourly (**A**) and daily (**B**) lifting condensation level at the HR_01_ (left) and HR_02_ (right) monitoring stations.

The distribution of particulate matter concentrations was negatively affected when vertical air dispersion was limited, particularly under conditions of a low lifting condensation level (LCL). As shown in Fig. 6, at the HR_02_ monitoring station, the average daily LCL followed a distinct pattern: after 9:00, the LCL began to rise steadily from approximatively 250 m, reaching a peak of around 1200 m by 16:00 in the afternoon. Following this, it gradually decreased during the evening and overnight, eventually dropping to approximately 400 m.

**Fig. 7:**
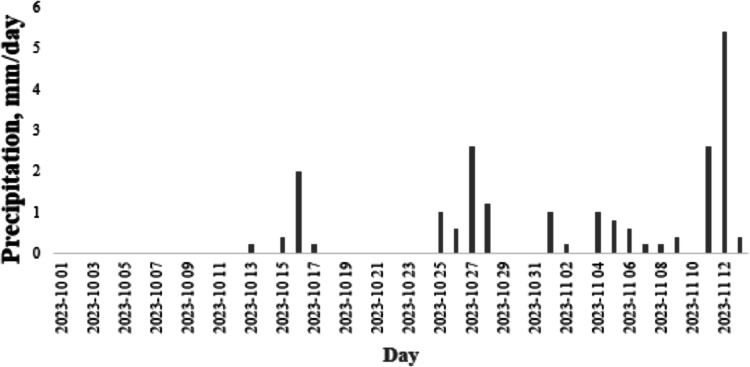
Evolution of the precipitation quantity during the monitoring period.

The average daily lifting condensation level (LCL) was measured at two monitoring stations. LCL values showed high variability, and on average, were higher on non-rainy days (Fig. 6). In the final week of the monitoring period, increased precipitation levels corresponded with a lower average LCL compared to the first week (Fig. 7).

### Studying the atmospheric washout effect of precipitation

During the study period, daily particulate matter concentrations measured in the city center were analyzed in relation to precipitation levels.

As shown in Table 2, the evolution of particulate matter concentrations was strongly influenced by the amount of precipitation. The highest particulate matter concentrations were observed on dry days, with an average TSP concentration of 98.07 μg m^–3^. In contrast, the lowest concentrations were recorded during heavy rainfall, averaging 35 μg m^–3^. Intermediate cases revealed a noticeable difference in aerosol concentrations measured during and after light to moderate rainfall intensities. These findings support the existence of an atmospheric cleansing effect where precipitation effectively reduces pariculate matter levels.

**Table 2:** Evolution of aerosol concentrations as a function of precipitation.

		PM_1.0_	PM_2.5_	PM_4.0_	PM_10_	TSP	Prec. mm
Without rain N = 7	Mean	**17.79**	**29.39**	**49.38**	**85.84**	**98.07**	**0.00**
	Stdev	5.1	8.0	12.9	22.6	26.1	
Low rainfall intensity (0.2–0.4 mm) N = 1	Mean	**9.34**	**15.12**	**24.25**	**40.40**	**46.09**	**0.40**
	Stdev	*	*	*	*	*	*
Moderate rain intensity (0.4–3.9 mm) N = 3	Mean	**11.35**	**18.57**	**30.59**	**53.18**	**61.07**	**0.60**
	Stdev	8.46	13.64	22.94	40.98	46.94	0.60
Heavy rain intensity (0.4–3.9 mm) N = 3	Mean	**9.43**	**14.07**	**19.64**	**30.39**	**35.01**	**6.67**
	Stdev	11.49	16.08	20.69	29.91	34.31	4.11

Significant values are in bold.

### Measured data versus monitoring station data

PM_10_ measurement were conducted over a two-week period (from 31.10.2023 to 13.11.2023), between 7:00 and 19:00 daily, and compared with hourly PM_10_ data from the HR_02_- LSPM_10_ monitoring station. It is important to note that the distance between the city center and the HR_02_ monitoring station, located on the southeastern edge of the city, is 1.1 km, with an average altitude difference of 23 m.

**Fig. 8:**
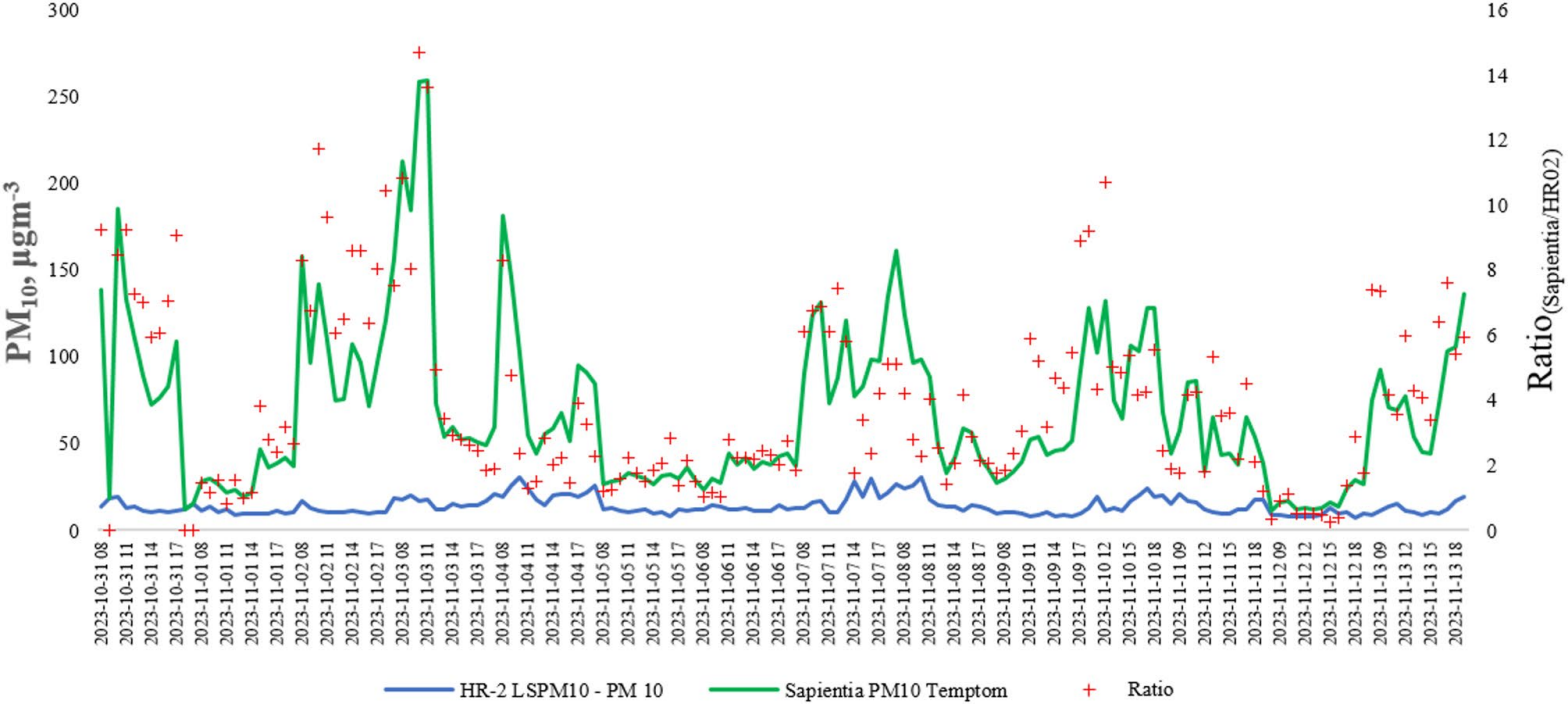
Comparison of PM_10_ concentrations between the city center and the HR_02_ monitoring station.

A significant discrepancy was observed between the two datasets. Out of 161 h of compared data, 150 cases showed higher particulate concentrations in the city center than at the HR_02_ station. In the few instances where concentrations were lower in the city center, the difference was minimal, averaging only 0.43%. Conversely, in the vast majority of cases (93.16%), the city center exhibited higher levels of particulate matter pollution, with concentrations averaging 3.27 times higher than those recorded at the HR_02_ station (Fig. 8.).

### Normality test and Spearman correlation analysis

**Fig. 9:**
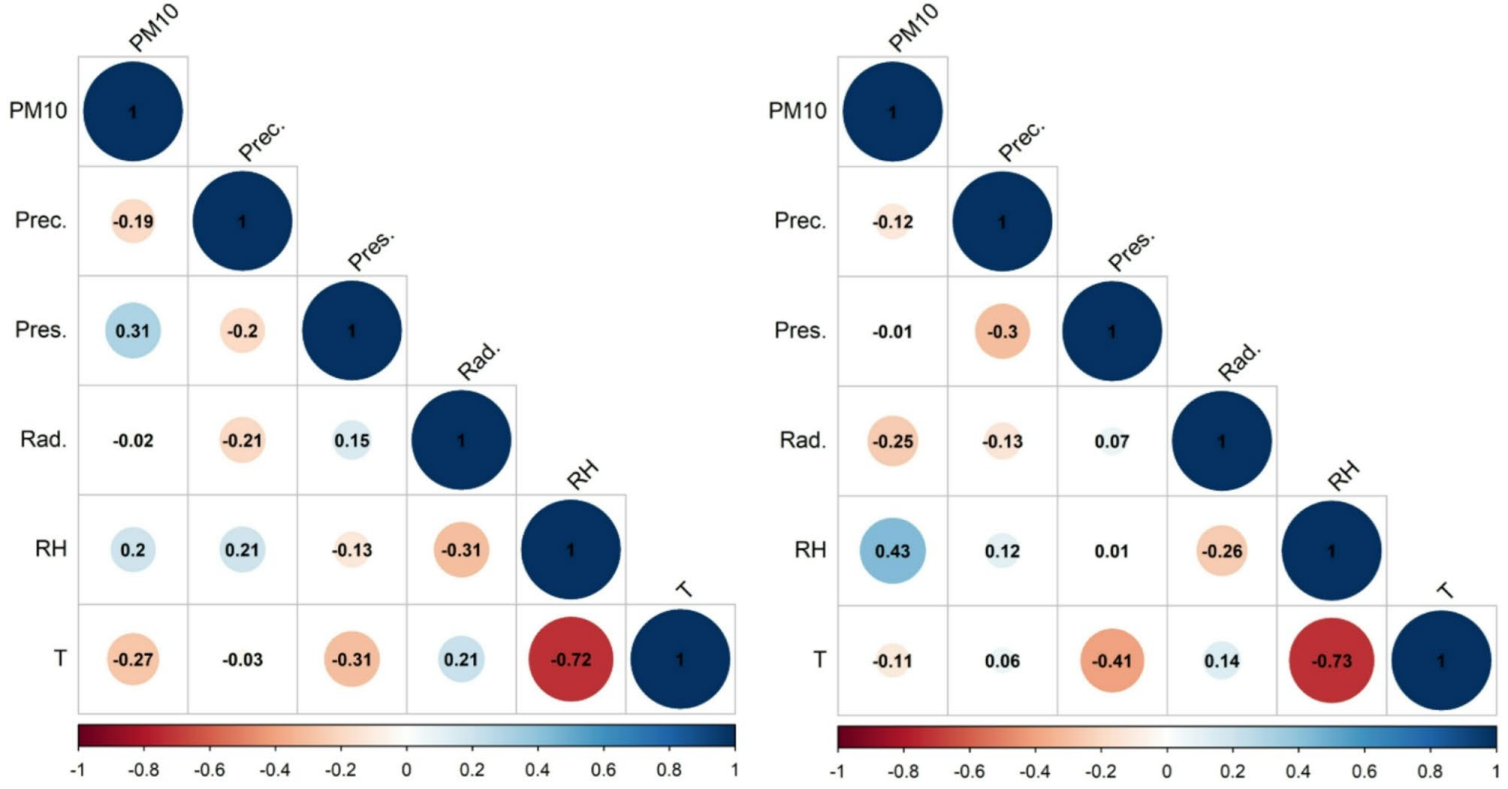
Correlation analysis with data from the city center (left) and monitoring station HR_02_ (right).

According to the normality test, the sample did not follow a normal distribution, which required the use of non-parametric methods, such as Spearman’s rank correlation, to explore relationships between variables. A histogram of the data is provided in Figure S1 in the appendix. The correlation coefficients between the measured particulate matter concentrations and the meteorological parameters are presented in Fig. 9. For the city center, 161 h of data were analyzed, whereas for the HR_02_ monitoring station, 336 h of data were available over the study period. The highest Spearman correlation coefficient was found between relative humidity and air temperature, with values of r = – 0.72 and – 0.73 for the two measuring points, respectively. This strong negative correlation reflects the natural inverse relationship between relative humidity and air temperature, as higher relative humidity is often associated with cooler temperatures. In the city center, a significant positive correlation was observed between PM_10_ and air pressure (r = 0.31) and between PM_10_ and relative humidity (r = 0.2), suggesting that specific meteorological conditions contribute to the accumulation of particulate matter. Relative humidity may enhance the hygroscopic growth of particles, increasing their mass and measured PM_10_ concentrations. At the HR_02_ monitoring station, the highest correlation was observed between relative humidity and PM_10_ concentration (r = 0.43). This indicates that relative humidity has a stronger influence on particulate matter at this site, possibly due to variations in local sources, land use, or microclimatic conditions.

### Air pollution concentration evolution as a function of traffic intensity

The increase in traffic intensity during the early morning hours showed a strong correlation with rising particulate matter concentrations. The lowest TSP levels were recorded at all sampling sites in the early hours (6:30 AM), while the maximum TSP concentration occurred between 7:30 and 8:30 AM, corresponding to a period when traffic intensity was two to three times higher than at the initial measurement. At Freedom Square, the TSP concentration during peak traffic (7:45–8:30 AM) was 2.13 times higher than the level measured at 6:30 AM (Table 3).

**Table 3:**
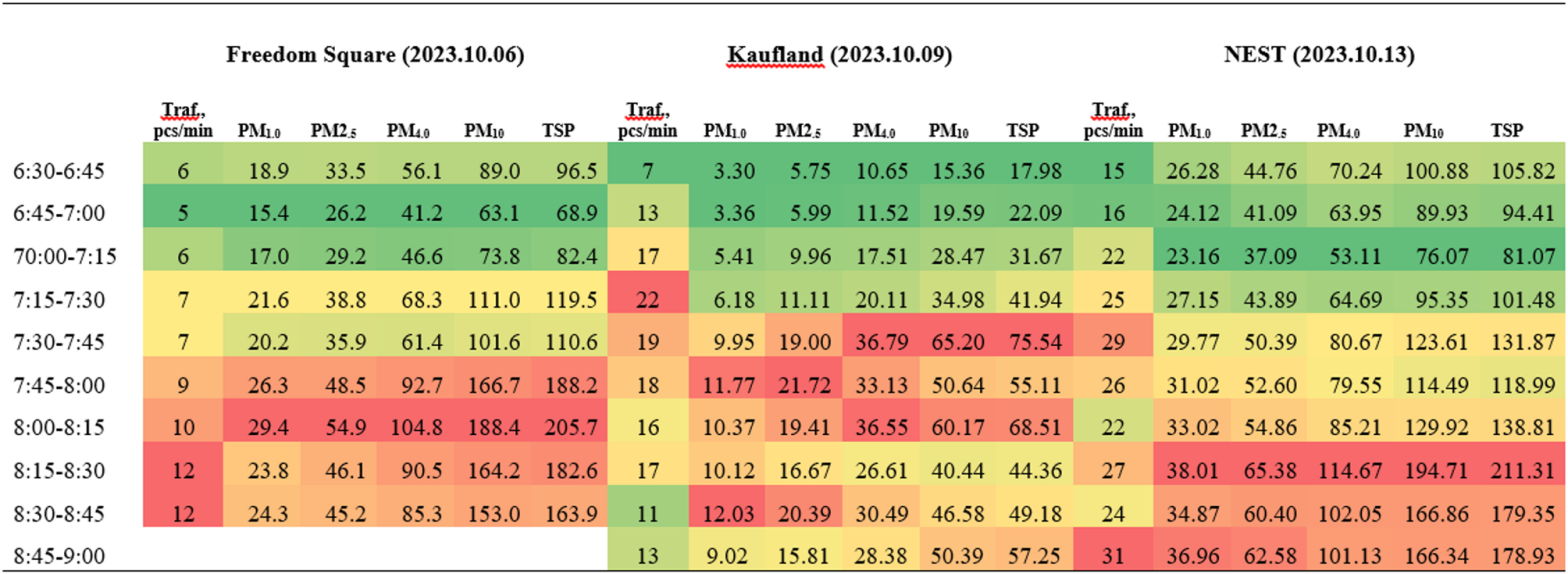
Traffic intensity and PM concentration variation during the morning period.

Around the Kaufland supermarket, the concentration of TSP ranged from a minimum of 17.98 μg m^–3^ to a maximum of 75.54 μg m^–3^, representing a 4.2 fold increase. The maximum traffic intensity occurred between 7:15–8:00 AM, with the highest TSP concentration (75.54 μg m^–3^) recorded between 7:30 and 7:45 AM. The difference between the highest and lowest traffic intensities was a factor of 3. Near the Nest shopping center, both the maximum TSP concentration and peak traffic intensity occurred between 8:15 and 9:00 AM, with a twofold difference between the two values.

### Study of the health effects of particulate matter at the monitoring station

According to the National Environmental Protection Agency’s report, the average concentration of PM_2.5_ in 2023 at the HR_02_ monitoring station was 21 μg m^–3^, more than twice the annual permissible limit of 10 μg m^–3^. Similarly, the PM_10_ concentration averaged 27 μg m^–3^ exceeding the annual limit of 20 μg m^–3^. The relative risk (RR) for all-cause mortality can be determined as a function of annual PM_10_ concentration. In 2023, the *RR* increase for all-cause mortality in the studied city was 14.6%. The table below includes possible annual concentrations ranging from 5 to 50 μg m^–3^. The calculated *RR* for cardiovascular disease (CVD) was *RR* = 1.303, and for lung cancer, the *RR* was 1.48 for PM_2.5_ (Table 4.).

**Table 4:** Calculate relative risks.

Summary table: relative risk assessment of short-term effects of particulate matter											
Annual concentration, μg m^-3^ (X)	PM_10_—Relative risk in relation to total mortality			PM_2.5_—Relative risk of death from cardiovascular disease				PM_2.5_—Relative risk of death from lung cancer			
PM_**10**_	RR	PM_10_	RR	PM_2.5_	RR	PM_2.5_	RR	PM_2.5_	RR	PM_2.5_	RR
5		28	1.155	5	1.065	28	1.360	5	1.099	28	1.584
6		29	1.164	6	1.091	29	1.367	6	1.139	29	1.597
7		30	1.174	7	1.114	30	1.374	7	1.175	30	1.609
8		31	1.183	8	1.134	31	1.381	8	1.207	31	1.621
9		32	1.192	9	1.153	32	1.387	9	1.237	32	1.632
10		33	1.202	10	1.17	33	1.394	10	1.265	33	1.644
11	1.008	34	1.212	11	1.186	34	1.400	11	1.291	34	1.655
12	1.016	35	1.221	12	1.201	35	1.406	12	1.315	35	1.666
13	1.024	36	1.231	13	1.215	36	1.412	13	1.338	36	1.676
14	1.033	37	1.241	14	1.228	37	1.418	14	1.359	37	1.687
15	1.041	38	1.251	15	1.240	38	1.424	15	1.380	38	1.697
16	1.049	39	1.261	16	1.252	39	1.429	16	1.399	39	1.707
17	1.058	40	1.271	17	1.263	40	1.435	17	1.418	40	1.717
18	1.066	41	1.281	18	1.273	41	1.44	18	1.436	41	1.726
19	1.075	42	1.292	19	1.284	42	1.446	19	1.453	42	1.736
20	1.083	43	1.302	20	1.293	43	1.451	20	1.47	43	1.745
21	1.092	44	1.313	21	1.303	44	1.456	21	1.486	44	1.754
22	1.101	45	1.323	22	1.312	45	1.461	22	1.501	45	1.763
23	1.11	46	1.334	23	1.320	46	1.466	23	1.516	46	1.772
24	1.119	47	1.344	24	1.329	47	1.47	24	1.53	47	1.781
25	1.127	48	1.355	25	1.337	48	1.475	25	1.544	48	1.789
26	1.137	49	1.366	26	1.345	49	1.48	26	1.558	49	1.798
27	1.146	50	1.377	27	1.352	50	1.484	27	1.571	50	1.806

## Discussion

The findings of this research offer valuable insights into the intricate dynamics of particulate matter (PM_1_, PM_2.5_, PM_4_, PM_10_, and total suspended particles TSPs) concentrations in urban environment, along with key factors influencing their variability. The results emphasize the multifaceted and complex nature of air pollution, shaped by both anthropogenic activities and natural phenomena (meteorological conditions). Our observations revealed notable spatial and temporal variations in particulate matter concentrations across multiple locations in Miercurea Ciuc. Significant elevations in PM concentrations during weekday mornings, compared to weekday afternoons highlight the influence of morning rush hour traffic on urban air quality. This finding aligns with existing literature, which identifies traffic emissions as a primary source of urban particulate matter. The observable reduction in PM concentrations during weekends suggests that decreased vehicular activity plays a crucial role in improving o air quality on non-working days^14–17^. Furthermore, the essential differences in PM concentration fluctuations between weekdays and weekends underscore altered traffic patterns on non-working days. The strong correlation identified between early morning rush- hour traffic and PM concentration increases underscores the pivotal role of vehicular emissions in driving urban air pollution. This highlighting the importance of implementing targeted traffic management strategies to reduce PM emissions during peak commuting hours and address air quality concerns effectively^18^.

Our examination of climatological parameters revealed intriguing trends. Specifically, during rainy periods, aerosol concentrations approached baseline values due to the cleansing effect of precipitation. In contrast, days without precipitation were associated with higher aerosol levels. This finding underscore the complex interaction between weather conditions and PM concentrations, suggesting that effective air quality management should consider meteorological forecasts and incorporate adaptive strategies to mitigate pollution levels in response to changing weather conditions^19^.

The comparison between continuous PM_10_ concentrations measured in the city center and hourly PM_10_ concentrations monitored by the Environmental Protection Agency (HR_02_) revealed substantial disparities, with the city center consistently showing higher PM_10_ concentrations. This finding underscores the localized nature of urban air pollution and highlight the critical need for localized monitoring and targeted mitigation efforts. The elevated concentrations in the city center can likely be attributed to factors such as denser traffic, higher population density, and the urban heat island effect, all of which can exacerbate pollution levels^20,21^.

These findings contribute significantly to our understanding of the spatiotemporal dynamics of particulate matter pollution in urban environments, highlighting the complex interplay between traffic patterns, climatological factors, and spatial heterogeneity. By shedding light of these factors, our study provide valuable insights for evidence-based policymaking and urban planning initiatives aimed at improving air quality and protecting public health in urban areas^22^.

To effectively improve air quality, local governments should implement comprehensive strategy that includes traffic management, urban planning, real-time monitoring, public engagement, and regulatory measures.

Traffic management can be optimized through dynamic congestion pricing during peak hours, with resulting revenues reinvested into sustainable transportation infrastructure. Expanding and modernizing public transit with electric buses, increasing service frequency, and offering subsidized fares can encourage higher usage. Furthermore, incentives like tax rebates, expanded EV charging infrastructure, and preferential parking can promote electric vehicle adoption. Urban planning should prioritize green spaces, rooftop gardens, and urban forests to mitigate the urban heat island effect and naturally filter air. Zoning regulations can separate industrial and residential areas, while promoting mixed-use developments encourages walking and cycling, reducing reliance on vehicles. Localized air quality monitoring through low-cost sensors in schools, hospitals, and neighborhoods will provide real-time data for targeted interventions. Integrating this data with traffic control systems can help manage traffic flow and inform timely public health advisories. Weather-informed strategies should incorporate predictive models that combine meteorological data with pollution sources to forecast air quality trends. Flexible protocols can adjust industrial emissions and traffic flow in response to forecasted pollution spikes. Public awareness campaigns are essential to engage communities. Educational programs on pollution risks and personal protection measures can be rolled out in schools and health facilities. Mobile apps and online platforms can provide real-time air quality data and health advisories while encouraging citizen reporting of pollution sources. Cross-sector collaboration with private companies and research institutions can drive innovation in pollution control technologies. Regional cooperation with neighboring municipalities can address cross-border pollution and standardize air quality regulations. Finally, a strong regulatory framework with stricter emissions standards, regular inspections, and penalties for violations is key. Financial incentives, like grants and tax breaks, can support businesses adopting cleaner technologies and sustainable practices.

In summary, the study provides a comprehensive analysis of the temporal and spatial patterns of particulate matter pollution in an urban setting, emphasizing the significant role of traffic emissions, weather conditions, and localized factors. By advancing our understanding of these dynamics, the research supports the development of targeted, effective strategies for improving air quality and protecting public health in urban environments. Future research should continue to explore the interactions between anthropogenic activities and natural processes to refine and enhance air quality management approaches^23^.

## Conclusions

This study provided a comprehensive analysis of particulate matter (PM_1_, PM_2.5_, PM_4_, PM_10_, and TSP) concentrations in Miercurea Ciuc, examining their spatial and temporal distribution in relation to road traffic intensity and meteorological parameters. The results revealed clear temporal patterns, with significantly elevated PM concentrations during weekday mornings compared to afternoons, mainly driven by rush hour traffic. In contrast, weekend levels were lower and more stable, reflecting reduced vehicular activity. These findings highlight the dominant role of traffic emissions in shaping urban air quality. Furthermore, aerosol levels declined notably during rainy periods, confirming the cleansing effect of precipitation.

The comparison between local city center measurements and those from the Environmental Protection Agency monitoring station (HR_02_) demonstrated substantial discrepancies, with inner-city PM_10_ levels averaging over three times higher. This underscores the localized nature of air pollution and the limitations of relying solely on centralized monitoring systems.

The study emphasizes the need for integrated and context-specific air quality management. Traffic management strategies—such as congestion pricing, improved public transit, and promotion of electric vehicles—can help reduce emissions during peak hours. Urban planning interventions, including the development of green spaces and reduction of urban heat islands, can further support air purification. Establishing a network of localized air quality sensors would provide real-time, high-resolution data to inform targeted mitigation actions. Additionally, integrating weather forecasting into air quality planning could help anticipate pollution peaks and adapt responses accordingly. Public education campaigns are essential to raise awareness about pollution sources, health impacts, and behavioral changes that can contribute to cleaner air.

In conclusion, this research contributes valuable insights into the complex dynamics of urban particulate pollution. It affirms the multifaceted impact of human activity and weather conditions on air quality, and provides a foundation for evidence-based policy decisions aimed at safeguarding public health and fostering sustainable urban environments. Future studies should continue exploring these interactions to refine pollution control efforts across diverse urban contexts.

## Limitation

A Teptom PMD immersive instrument was used for clear fog-free weather. A comparison of fine and coarse particulate matter (PM_10_ particulate matter in the city center vs. that at the regional monitoring station LSP) was feasible, and hourly gravimetric measurements were not available at the time of the study. PM concentrations measured at different locations in the city were measured using the same measuring device within a ± 1 h time difference.

## Electronic supplementary material

Below is the link to the electronic supplementary material.

Supplementary Material 1

## Data Availability

Availability of data and material: The datasets used and analyzed during the current study are available from the corresponding author upon reasonable request.
