# Supplementary material for: Examination of particulate matter concentrations of the outdoor air of Miercurea Ciuc, Romania
Source: Sci Rep. 2025 Jul 2;15:23229. doi: 10.1038/s41598-025-04528-w (PMC12223154; doi:10.1038/s41598-025-04528-w)
Supplement: Supplementary file 1 — Supplementary Material 1 [file 41598_2025_4528_MOESM1_ESM.docx]

**Appendix**

S1.Table: Features of the Temptom PMD 351 device

| Measured fractions | PM_1.0_, PM_2.5_, PM_4.0_, PM_10_,TSP |
| --- | --- |
| Measuring range | 0~1000 μg m^-3^ |
| Accuracy | ±10% |
| Resolution | 0.1 μg m^-3^ |
| Measurement principle | Light scattering technique |
| Light source | 50mW, 780nm |
| Sampling time | 1 min |
| Volume flow | 2.83 L min^-1^ |
| Display | 4.0" TFT LCD screen |
| Communication | USB /RS-232 |
| Memory | 2,000,000 measurements |
| Battery | Rechargeable lithium battery |
| Charging time | 3.5 hour |
| Operating time | 8 hour |
| Operating Temperature | 0~50 ℃ |
| Storage temperature | -20~60 ℃ |
| Monitor dimension | 170×110×48 mm |
| Mass | 850g |

**Figure S1.** PM_10_ and the environmental parameters distribution
